# Circulating progenitor and angiogenic cell frequencies are abnormally static over pregnancy in women with preconception diabetes: A pilot study

**DOI:** 10.1371/journal.pone.0172988

**Published:** 2017-03-09

**Authors:** Patricia D. A. Lima, Zhilin Chen, Aysha Tayab, Malia S. Q. Murphy, Jessica Pudwell, Graeme N. Smith, B. Anne Croy

**Affiliations:** 1 Department of Biomedical and Molecular Science, Queen’s University, Kingston, Ontario, Canada; 2 Department of Obstetrics and Gynecology, Queen’s University, Kingston, Ontario, Canada; Centro Cardiologico Monzino, ITALY

## Abstract

Type 1 and 2 diabetes decrease the frequencies and functional capacities of circulating angiogenic cells (CAC). Diabetes also elevates gestational complications. These observations may be interrelated. We undertook pilot studies to address the hypothesis that preconception diabetes deviates known gestational increases in CACs. Cross-sectional study of type 1 diabetic, type 2 diabetic and normoglycemic pregnant women was conducted at 1^st^, 2^nd^, and 3^rd^ trimester and compared to a 6mo postpartum surrogate baseline. Circulating progenitor cells (CPC; CD34+CD45dimSSlow) and CACs (CD34+CD45dimSSlow expressing CD133 without or with KDR) were quantified by flow cytometry and by colony assay (CFU-Hill). In pregnant normoglycemic women, CD34+CD45dimSSlow cell frequency was greater in 1^st^ and 3^rd^ trimester than postpartum but frequency of these cells was static over type 1 or 2 diabetic pregnancies. Type 1 and type 2 diabetic women showed CACs variance versus normal controls. Type 1 diabetic women had more total CD34+KDR+ CACs in 1^st^ trimester and a higher ratio of CD133+KDR+ to total CD133+ cells in 1^st^ and 2^nd^ trimesters than control women, demonstrating an unbalance in CD133+KDR+ CACs. Type 2 diabetic women had more CD133+KDR+ CACs in 1^st^ trimester and fewer CD133+KDR- CACs at mid-late pregnancy than normal pregnant women. Thus, pregnancy stage-specific physiological fluctuation in CPCs (CD34+) and CACs (CD133+KDR+ and CD133+KDR-) did not occur in type 1 and type 2 diabetic women. Early outgrowth colonies were stable across normal and diabetic pregnancies. Therefore, preconception diabetes blocks the normal dynamic pattern of CAC frequencies across gestation but does not alter colony growth. The differences between diabetic and typical women were seen at specific gestational stages that may be critical for initiation of the uterine vascular pathologies characterizing diabetic gestations.

## Introduction

Various circulating, bone marrow-derived, vasculotrophic cell types support vessel repair and neoangiogenesis [1]. Circulating angiogenic cells (CACs), originally termed circulating endothelial progenitor cells (EPCs), were considered a single lineage that differentiated into mature endothelium. EPCs were phenotypically defined as Lin-CD34+CD45- or CD45dim and reactive with either CD133 or KDR (Kinase insert Domain Receptor; VEGFR2 or CD309). These populations gave rise to two types of colonies in culture [1,2]. Although early outgrowth colonies, known as “colony forming units (CFU)-Hill” grew with endothelial cell-like morphology, they are now known to include myeloid cells (macrophages and monocytes) and to have frequent T cell contamination. Cells forming these mixed colonies however, are still thought to promote angiogenesis and vascular repair [3]. Early outgrowth colonies are used to predict patient cardiovascular risk [4]. The other colony type (later outgrowth colonies or outgrowing endothelial cells), were much rarer and displayed properties resembling endothelial cell lines. Later outgrowth colonies emerge only after long time culture and it uncertain whether this population differentiates *in vivo*. Concepts regarding EPCs have been considerably refined. Heterogeneous circulating vasculotrophic cells are now recognized, most of which localize to perivascular spaces rather than differentiating into endothelium. CPC and CAC represent cell populations enriched in monocytes that exert their angiogenic effects via paracrine mechanisms. Here we addressed the dual impacts of diabetes and pregnancy on CPCs (CD34+CD45dimSSlow), CACs (CD34+CD45dimSSlow expressing CD133 and/or KDR) and on early outgrowth colonies.

Healthy pregnancy is a physiological state heavily dependent upon maternal utero-placental and systemic angiogenesis and vascular remodeling. Early maternal angiogenesis is driven by nidatory hormones and conceptus implantation. Subsequently, angiokines are secreted into the maternal circulation by the conceptus-derived placenta [5–8] and by maternal decidual leukocytes [9,10]. In healthy pregnancies, CPCs (CD34+) and CACs (CD34+KDR+ and CD133+KDR+) are reported to increase by 2^nd^ and into 3^rd^ trimester then decline to nulligravida levels by 48h postpartum [11,12,13]. Little is known regarding CPC or CAC functions during maternal uterine or systemic vascular remodeling and expansion that typifies normal pregnancy. It is reported that the menstrual cycle and menopause alter CD34+KDR+ CACs frequency via estrogen-based mechanisms [14]. In the common pregnancy complications of preeclampsia [12,15] and new onset gestational diabetes [13,16], CAC frequencies are lower than in normoglycemic pregnant women and colony growth is slower. Preeclampsia, an acute, multi-organ vascular syndrome of late pregnancy, is associated with high maternal plasma sFLT1 [17], a decoy receptor for vascular endothelial growth factor (VEGF). Pregnant diabetic women have elevated preeclampsia risk, regardless of their underlying diabetic etiology [18–20]. No publications report CACs levels during pregnancy in normotensive women with established preconception diabetes.

Non-pregnant, type 1 and type 2 diabetic individuals exhibit vascular and endothelial dysfunctions [21, 22]. The two “hits” of reduced CAC frequency and impaired regenerative capacity [23–27] are thought to compromise vascular health [26– 28], predict cardiovascular disorders [20, 27, 29] and limit vascular repair. We hypothesized that CPC and CAC frequencies would increase across normal pregnancy when compared their frequencies in non-pregnant women. We further postulated that preconception diabetes would diminish these gains, even in women with regular physician-supervised and individually- tailored diabetic management, and this reduction would contribute to the maternal and fetal complications characteristic of diabetic pregnancies. Our aim was to quantify CPC and CACs across pregnancy (1^st^, 2^nd^, and 3^rd^ trimester) in type 1 and type 2 diabetic women and matched normoglycemic women carrying singleton fetuses and attending a tertiary care obstetrical diabetes clinic. Postpartum (~6 months) was used as a surrogate time point for preconception, baseline CPC and CAC values, since our patient referrals follow conception. Differences in CPC and CAC frequencies were documented between pregnant diabetic women and women experiencing typical pregnancies and between pregnant type 1 and pregnant type 2 diabetic patients.

## Material and methods

### Patients and recruitment

Pregnant and postpartum women with type 1 diabetes (n = 32) or type 2 diabetes (n = 28), and healthy age and gestation stage-matched controls (n = 49) were recruited at Kingston General Hospital (Kingston, ON, Canada). Diabetic patients regularly attended the diabetic pregnancy clinic for supervised care. Exclusion criteria were more than one fetus or previous diagnosis or treatment of hypertension, polycystic ovarian syndrome, cancer and/or autoimmune disease other than diabetes. Patients who provided written consent were blind encoded to the investigators and no personal identifying information was recorded. Protocols were pre-approved by the Queen’s University Health Sciences and Affiliated Teaching Hospitals Research Ethics Board (ANAT-030-10). Blood was collected from different participants at 6–13 weeks (1^st^ trimester), 18–26 weeks (2^nd^ trimester), 28–36 weeks (3^rd^ trimester) or postpartum (~6 months) into acid citrate dextrose anti-coagulant by venipuncture (BDVacutainer, BD Bioscience; Mississauga, ON, Canada). Mononuclear cells were immediately isolated by centrifugation (400 g; 30 min; 4°C) using Histopaque 1077 (Sigma Aldrich, St. Louis, MO, USA), recovered, washed twice (PBS; 600 g; 10 min; 4°C), enumerated using a hemocytometer then aliquoted for flow cytometry or CFU- Hill culture.

### Flow cytometry

The modified protocol of the International Society of Hematology and Graft Engineering (ISHAGE) was used to examine 1x10^5^ washed cells that had been incubated (30 min; 4°C in dark) in 100 uL of PBS containing 1% BSA (Sigma Aldrich, Oakville, ON, Canada), 2% heat-inactivated human serum (Life Technology, Canada; FACS buffer) and antibodies directly conjugated to fluorochromes: anti-CD45 Phycoerythrin Cyanin 7 (Biolegend, San Diego, CA, USA), anti-CD34 FITC (BD Pharmigen, San Diego, CA, USA), anti-CD133 Phycoerythrin (Macs Miltenyl Biotec, Alburn, CA, USA) and anti-KDR PerCp (R&D System, Minneapolis, MN, USA) and their respectively and isotype controls in concentrations suggested by the manufacturer (Table 1). After washing in PBS, cells were fixed 2% paraformaldehyde and analysed (Beckman Coulter Cytomics FC500; Beckman Coulter, Inc., Mississauga, ON, Canada). Post-acquisition compensation and analyses were performed using Kaluza software (Beckman Coulter, Inc.).

**Table 1:** Antibody and isotype information.

Antibody and Isotypes	Fluorochrome	Company	Catalogue number
Anti-CD34	FITC	**BD Pharmigen**	**S55821**
Anit-CD45	Phycoerythrin Cyanin 7	**Biolegend**	**304016**
Anti-CD133	Phycoerythrin	**Macs**	**130-090-826**
Anti-KDR	PerCP	**R&D System**	**FAB357C**
IgG1	FITC	**Biolegend**	**981802**
IgG1	Phycoerythrin Cyanin 7	**Biolegend**	**400126**
IgG1	Phycoerythrin	**Biolegend**	**400112**
IgG1	PerCP	**Biolegend**	**400148**

### Early outgrowth colonies (CFU-Hill assay)

For clonogenic assays, 5x10^6^ washed mononuclear cells were resuspended in 1.5mL CFU-Hill Basal Medium (Stem Cell Technologies Inc., Vancouver, Canada) with CFU-Hill proliferation supplement (Stem Cell Technologies Inc.) according to supplier instructions, plated on fibronectin-coated 35 mm dishes (Biocoat BD Bioscience, Mississauga, ON, Canada) and incubated (37°C; 5% CO2). After pre-plating depletion (48 h) of adherent macrophages and mature endothelial cells, non-adherent cells were collected, re-plated at 2x10^6^ cells per well on fibronectin-coated 4-well chamber slides (Biocoat BD Bioscience) and cultured 72 h. Then, medium was removed, wells were washed in PBS and Giemsa stained (Sigma-Aldrich, Oakville, ON, Canada). Colonies, defined as a central core of round cells with peripheral elongated sprouting cells, were enumerated.

### Statistical analyses

Continuous variables were tested for normality using Kolmogorov-Smirnov test. Non-parametric Kruskal-Wallis one-way analysis of variance test followed by Dunn’s multi-comparisons post-test was used to compare values across pregnancy and postpartum within the same group (control, type 1 diabetes or type 2 diabetes), the CFU-Hill data and patient clinical information (family wise significance and confidence levels ≤ 0.05). Specific comparisons between control, type 1 diabetes and type 2 diabetes patients were performed using Mann-Whitney U- Test (two-tailed and 95% of confidence level). The ROUT method was used as outlier identifier (Q = 1%). Data are expressed as mean±SEM or percentage, unless otherwise stated.

## Results

Age, parity, number of term deliveries, abortions or living children did not differ between patient groups. More preterm deliveries occurred in type 2 diabetic patients than in controls with, 41.71% of type 2 diabetic women delivering preterm compared to 2.70% controls (Table 2). Preeclampsia was more frequent in type 1 diabetic women (28.54%) compared to controls (0%). Weight gain, systolic and diastolic blood pressures and blood glucose at 2^nd^ and 3^rd^ trimester in diabetic patients matched controls. Mean HbA1C (%) was between 6.50% and 7.20% (levels >6.5% indicate diabetes) in type 1 and type 2 diabetic patients, target values recommended by the Canadian Diabetes Association (Table 2). Microalbumin to creatinine ratios (M/C) in diabetic patients were >26.63 ± 17.90 mg/mmol (normal female values < 2 mg/mmol; >30 mg/mmol defined as microalbuminuria).

**Table 2:** Maternal information.

Demographic				
	**Control (36)**	**Type 1 diabetes (12)**	**Type 2 diabetes (17)**	***P***
**Age**	29.89 (±0.68)	27.17 (±1.30)	31.41 (±1.22)	0.101
**Obstetric History**				
*Gravida*	2.02 (±0.23)	2.00 (±0.32)	2.76 (±0.31)	0.069
*Term Deliveries %*^†^	44.44	33.33	41.17	0.675
*Preterm Deliveries %*^†^	*2*.70**	***25*.*00***	*41*.71**	***0*.*001***
*Abortions %*^†^	22.24	25.00	41.17	0.343
*Living Children*	0.75 (±0.18)	0.58 (±0.22)	1.176 (±0.24)	0.117
*Preeclampsia%*^†^	0**	*28*.54**	10.00	***0*.*003***
**Clinical**				
	**Control**	**Type 1 diabetes**	**Type 2 diabetes**	***P***
**Weight gain (Kg)- Pregnant—non-pregnant weight**				
*2*^*nd*^ *trimester*	4.90 (±1.93)	11.29 (±2.85)	7.03 (±2.2)	0.219
*3*^*rd*^ *trimester*	11.72 (±2.60)	10.85 (±4.45)	12.24 (±2.42)	0.779
**Systolic Blood Pressure (mmHg)**				
*2*^*nd*^ *trimester*	114.70 (±3.71)	110.00 (±3.65)	123.00 (±3.83)	0.103
*3*^*rd*^ *trimester*	111.80 (±2.80)	11.60 (±5.87)	123.70 (±7.45)	0.228
**Diastolic Blood Pressure (mmHg)**				
*2*^*nd*^ *trimester*	75.00 (±1.52)	68.60 (±1.90)	67.30 (±2.42)	0.205
*3*^*rd*^ *trimester*	67.30 (±1.97)	69.20 (±2.49)	75.00 (±4.69)	0.387
**Blood Glucose (mmol/L)**				
*2*^*nd*^ *trimester*	5.32 (±0.11)	6.76 (±1.17)	6.93 (±0.65)	0.271
*3*^*rd*^ *trimester*	4.86 (±0.89)	6.83 (±1.38)	5.68 (±0.77)	0.348
**HbA1C**				
*2*^*nd*^ *trimester*	NAD	6.70 (±0.3) (%)	6.60 (±0.3) (%)	0.903
		50(±7.4) (mmol/mol)	49.40(±12.6) (mmol/mol)	0.928
*3*^*rd*^ *trimester*	NAD	7.20 (±0.6) (%)	6.50 (±0.3) (%)	0.333
		55.17 (±16.45) (mmol/mol)	47.33 (±9.7) (mmol/mol)	0.389
**Microalbumin:Creatinine ratio (mg/mmol)**				
*2*^*nd*^ *trimester*	NAD	69.93 (±60.29)	43.72 (±34.99)	0.697
*3*^*rd*^ *trimester*	NAD	26.63 (±17.90)	143.0 (±133.7)	0.539

Data are presented as mean± SEM or percentage. NAD- no abnormality detected; Normal ranges of glucose (3.3–5.6mmol/L), HbA1C (3.6–5.0% of Hb) and microalbumin:creatinine ratio (<2.0 mg/mmol).

^**†**^ Frequency of women within a group.

Significant values of p≤ 0.01 (**).

HbA1C conversion using: http://www.ngsp.org/convert1.asp.

**Fig 1:**
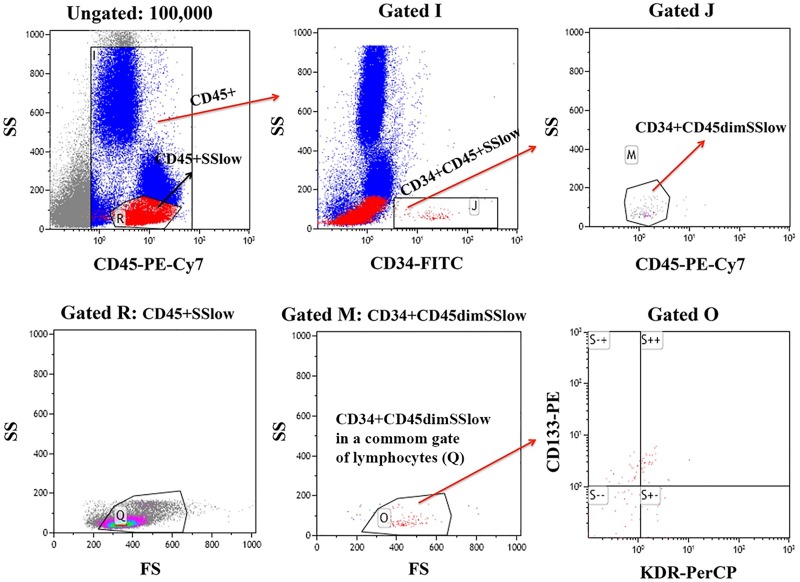
Gating strategy based on ISHAGE guidelines. Total CD45+ cells were gated, followed by Side Scatter (SS) *vs* CD34-FITC. The CD34+CD45+SSlow subset was selected (Gate J). A second SS *vs* CD45-PE-Cy7 gating was performed to select CD45dim cells (Gate M), and confirmed by a low Forward Scatter (R- a shared lymphocyte gate; Gate O). Analyzing CD34-FITC *vs* CD45-PE-Cy7 cells, a subset should be coincident with gate Q (lymphocyte). Expression of CD133 and/or KDR was assessed on CD34+CD45dimSSlow cells (gated O).

**Fig 2:**
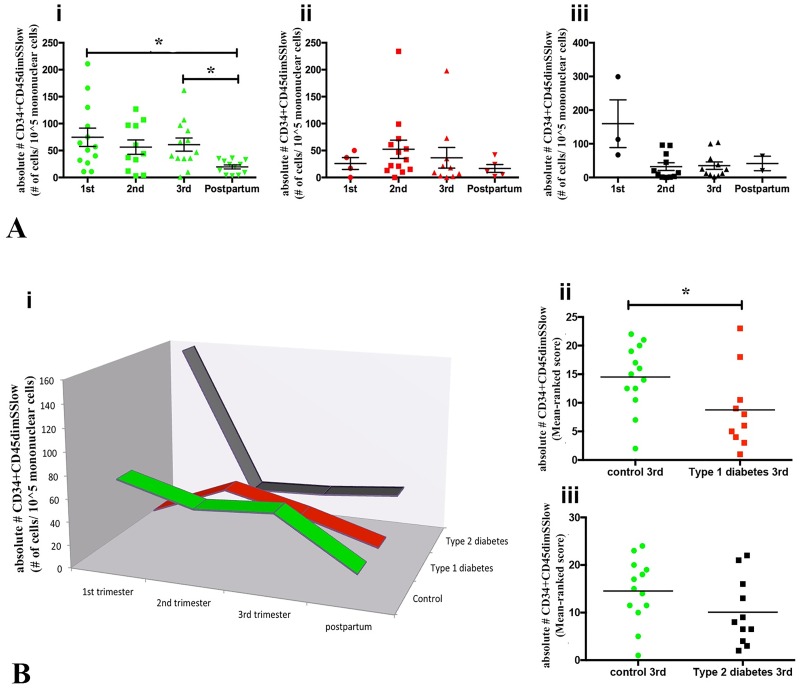
Total Circulating Progenitor Cell (CPC) analysis through pregnancy and postpartum in control, type 1 and type 2 diabetic women. **(A**) Absolute numbers of circulating CD45dimCD34+SSlow cells across pregnancy and postpartum from control (**i**), type 1 (**ii**) and type 2 (**iii**) diabetic women. In control women, CD45dimCD34+SSlow cells were significantly higher in the 1^st^ (p = 0.013) and 3^rd^ (p = 0.030) trimester compared to postpartum, but not in type 1 and type 2 diabetic women. (**B)** The 3D-line chart demonstrates the fluctuations in CD45dimCD34+SSlow cells over pregnancy and in a non-pregnancy state (postpartum) in control (green), type 1 (red) and type 2 (dark gray) diabetic pregnancies (**i)**, while (**ii**) and (**iii)** represent the absolute numbers of CD45dimCD34+SSlow cells per 10^5^ mononuclear cells from control (green), type 1 (red) (**ii**) and type 2 (black) (**iii**) diabetic women at 3^rd^ trimester. CD45dimCD34+SSlow cells were significantly more abundant in control compared to type 1 (p = 0.043), but not type 2 diabetic women (p = 0.130). Data in **A** were compared using Kruskal-Wallis test; **Bii** and **Biii** using Mann-Whitney test, considering p<0.05. Data in **Bii** and **Biii** are shown as a rank score graphic.

CPCs were defined as CD34+ cells that had low side scatter (SS) and CD45 expression (CD34+CD45dimSSlow). CACs were defined as CD34+CD45dimSSlow cells expressing CD133 or KDR (Fig 1). In control women, frequencies of circulating CD34+CD45dimSSlow cell in 1^st^ and 3^rd^ trimester were higher than at 6 months postpartum (p = 0.013 and p = 0.030, respectively). Second trimester frequency did not differ to postpartum (p = 0.116; Fig 2Ai). In contrast, CD34+CD45dimSSlow cell numbers were stable in type 1 and type 2 diabetic women across pregnancy and through postpartum (Fig 2Aii and 2Aiii). When circulating CD34+CD45dimSSlow cell numbers were compared among groups (Fig 2Bi; Table 3) within a gestational stage or postpartum, circulating CD34+CD45dimSSlow cells were significantly higher at 3^rd^ trimester of control pregnancies compared to type 1 diabetic women (p = 0.043; Fig 2Bii; Table 3). Type 2 diabetic women at 3^rd^ trimester also had fewer CD34+CD45dimSSlow cells compared to control women, but this difference was not statistically significant (p = 0.130; Fig 2Biii; Table 3).

**Table 3:** CPC and CAC numbers per 10^5^ blood mononuclear cells across pregnancy and postpartum.

**Circulating CD34+CD45dimSSlow cells**				**P value**		
	**Control**	**Type 1 diabetes**	**Type 2 diabetes**	**Control *vs type 1***	**Control *vs* type 2**	**Type 1 *vs* 2**
***1***^***st***^ ***trimester***	74.62 (±16.94)	26.00(±11.01)	159.7(±70.92)	0.097	0.139	0.057
***2***^***nd***^ ***trimester***	56.36 (±13.29)	11.01(±17.06)	32.27(±11.45)	0.659	0.112	0.245
***3***^***rd***^ ***trimester***	*61*.*00(±12*.18)*	*36*.*60(±19*.23)*	35.09(±11.05)	0.043	0.130	0.436
***Postpartum***	19.58 (±3.64)	16.80 (±7.34)	41.50(±21.50)	0.703	NSA	NSA
**Circulating CD34+133+KDR- cells**				**P value**		
	**Control**	**Type 1 diabetes**	**Type 2 diabetes**	**Control *vs type 1***	**Control *vs* type 2**	**Type 1 *vs* 2**
***1***^***st***^ ***trimester***	48.08 (±15.29)	2.00 (±2.00)	99.00(±95.50)	0.178	0.673	0.300
***2***^***nd***^ ***trimester***	48.78*(±13*.64)*	17.75 (±7.09)	*7*.*00 (±3*.02)*	0.074	0.028	0.625
***3***^***rd***^ ***trimester***	*39*.*58(±11*.64)**	16.29 (±9.67)	*3*.*50(±2*.30)**	0.152	0.004	0.775
***Postpartum***	2.66 (±2.42)	0.75 (±0.47)	0.50 (±0.50)	0.725	NSA	NSA
**Circulating CD34+CD133+KDR+ cells**				**P value**		
	**Control**	**Type 1 diabetes**	**Type 2 diabetes**	**Control *vs type 1***	**Control *vs* type 2**	**Type 1 *vs* 2**
***1***^***st***^ ***trimester***	*4*.*30 (±1*.50)*	13.00 (±9.00)	*31*.*33(±14*.62)*	0.091	0.032	0.400
***2***^***nd***^ ***trimester***	3.33 (±0.79)	23.25 (±12.68)	14.14 (±9.20)	0.278	0.447	0.213
***3***^***rd***^ ***trimester***	10.00(±2.47)	26.14 (±17.88)	9.70 ***(***±5.32)	0.853	0.369	0.348
***Postpartum***	1.88 (±0.45)	5.50 (±3.663)	3.50 (±1.50)	0.581	NSA	NSA
**Circulating CD34+CD133-KDR+ cells**				**P value**		
	**Control**	**Type 1 diabetes**	**Type 2 diabetes**	**Control *vs type 1***	**Control *vs* type 2**	**Type 1 *vs* 2**
***1***^***st***^ ***trimester***	4.53 (±2.83)	5.66 (±1.20)	16.67(±14.71)	0.075	0.519	0.800
***2***^***nd***^ ***trimester***	4.33 (±3.83)	4.75 (±2.17)	4.71 (±1.79)	0.153	0.184	0.630
***3***^***rd***^ ***trimester***	3.83 (±1.46)	4.71(±1.91)	5.80 (±2.00)	0.534	0.377	0.904
***Postpartum***	3.11(±0.67)	3.50 (±1.19)	2.00 (±1.00)	0.988	NSA	NSA
**Circulating CD34+CD133-KDR- cells**				**P value**		
	**Control**	**Type 1 diabetes**	**Type 2 diabetes**	**Control *vs type 1***	**Control *vs* type 2**	**Type 1 *vs* 2**
***1***^***st***^ ***trimester***	17.69 (±4.26)	14.00(±7.55)	12.67 (±4.97)	0.883	0.921	> 0.99
***2***^***nd***^ ***trimester***	11.67 (±4.15)	10.92(±3.04)	24.14 (±9.06)	0.958	0.423	0.308
***3***^***rd***^ ***trimester***	12.50 (±4.88)	*4*.*42(±2*.52)*	*19*.*40 (±5*.68)*	0.111	0.259	0.030
***Postpartum***	17.22 (±2.98)	10.75(±9.42)	35.50 (±24.50)	0.179	NSA	NSA

Mann-Whitney-U test (data not normally distributed: Kolmogorov–Smirnov). Data are shown as means of absolute numbers (±SEM). P values refer to comparisons between Control-type 1 diabetes; control-type 2 diabetes; or type1-type 2 diabetes.

Significance: p ≤ 0.05 (*), p≤ 0.01 (**).

Not statistically analyzed (NSA) due to low number of type 2 diabetes postpartum samples.

**Fig 3:**
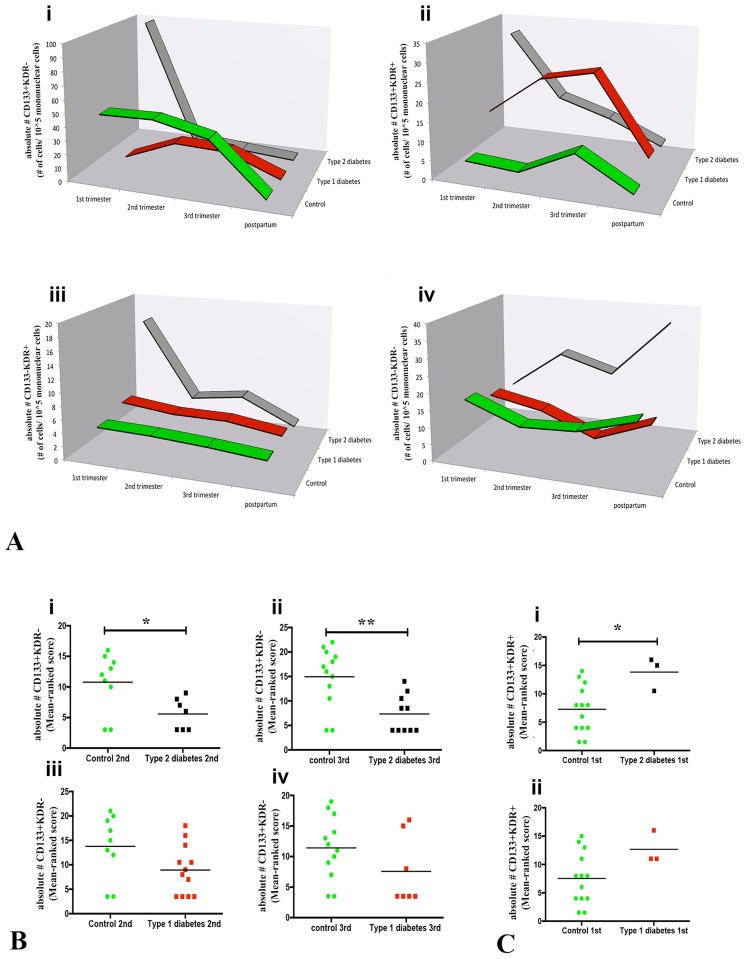
CAC (CD133+KDR-, CD133+KDR+, CD133-KDR+) analyses through pregnancy and postpartum in control, type 1 and type 2 diabetic women. **(A)** The 3D-line chart demonstrates fluctuations in the average of CD133+KDR- **(i)**, CD133+KDR+ (**ii)**, CD133-KDR+ (**iii**) and CD133-KDR- CACs (**iv**) over pregnancy and postpartum in control (green), type 1 (red) and type 2 (dark gray) diabetic women. (**B)** Punctual analysis comparing absolute numbers of CACs within specific gestational periods or postpartum demonstrated that CD133+KDR- CACs were lower in 2^nd^ (**i;** p = 0.028) and 3^rd^ (**ii**; p = 0.004) trimesters of type 2 diabetic (black) women compared to control (green). CD133+KDR- CACs appeared to be fewer in type 1 diabetic women 2^nd^ **(iii**; p = 0.074) and 3^rd^ (**iv**; p = 0.152) trimesters but the differences were not statistically significant versus controls. (**C)** CD133+KDR+ CAC numbers were significantly higher in 1^st^ trimester type 2 diabetic women (**i**; p = 0.032) than in controls. This difference was not present in type 1 diabetes although the overall pattern of type 1 diabetes fluctuations resembled that of type 2 diabetes (**ii**; p = 0.091). (**B)** and (**C)** data were compared using Mann-Whitney test, considering p<0.05; and they are shown in rank score graphic.

In control women, CD133+KDR- CACs appeared to be increased by pregnancy but this trend did not reach statistical significance compared to a non-pregnant state (6 months postpartum *vs* 1^st^ p = 0.082, *vs* 2^nd^ trimester p = 0.057 and *vs* 3^rd^ trimester p = 0.057). No pregnancy-linked elevation in CD133+KDR- CACs occurred in type 1 or type 2 diabetes (Fig 3Ai). Indeed, CD133+KDR- CACs from 2^nd^ and 3^rd^ trimesters of type 2 diabetic women were significantly rarer than in control women (p = 0.028 and p = 0.004, respectively; Fig 3Bi and 3Bii; Table 3). Similar statistical differences in CD133+KDR- cell frequency at 2^nd^ and 3^rd^ trimesters were not confirmed for type 1 diabetic women (p = 0.074 and p = 0.152, respectively; Fig 3Biii and 3Biv; Table 3).

CD133+KDR+ and CD133-KDR+ CACs were less abundant in the circulations of control and diabetic women than CD133+KDR- cells. Neither KDR+ CAC subset differed statistically across pregnancy and postpartum within a group (Fig 3Aii and 3Aiii; Table 3). Generally, diabetic women (type 1 and type 2) had more circulating CD133+KDR+ CACs than controls (Fig 3Aii; Table 3). CD133+KDR+ CACs were significantly more abundant in type 2 diabetic women at 1^st^ trimester compared to controls (p = 0.032; Fig 3Ci). This difference was not observed in 1^st^ trimester type 1 diabetic women (p = 0.091; Fig 3Cii). The CD133-KDR+ CAC subset was constant across pregnancy or postpartum within a group, and it did not significantly differ among groups (Fig 3Aiii; Table 3). CD34+ cells that did not express CD133 or KDR were also analyzed. Numbers of these hematopoietic progenitor cells did not vary across pregnancy and postpartum in any group (Fig 3Aiv; Table 3). Additionally, no differences were found comparing circulating CD34+CD133-KDR- cell numbers between diabetic women (type 1 or type 2) and control women (Table 3). At 3^rd^ trimester however, type 2 diabetic women had elevated CD34+CD133-KDR- cell numbers compared to type 1 diabetic women (p = 0.030; Table 3).

**Fig 4:**
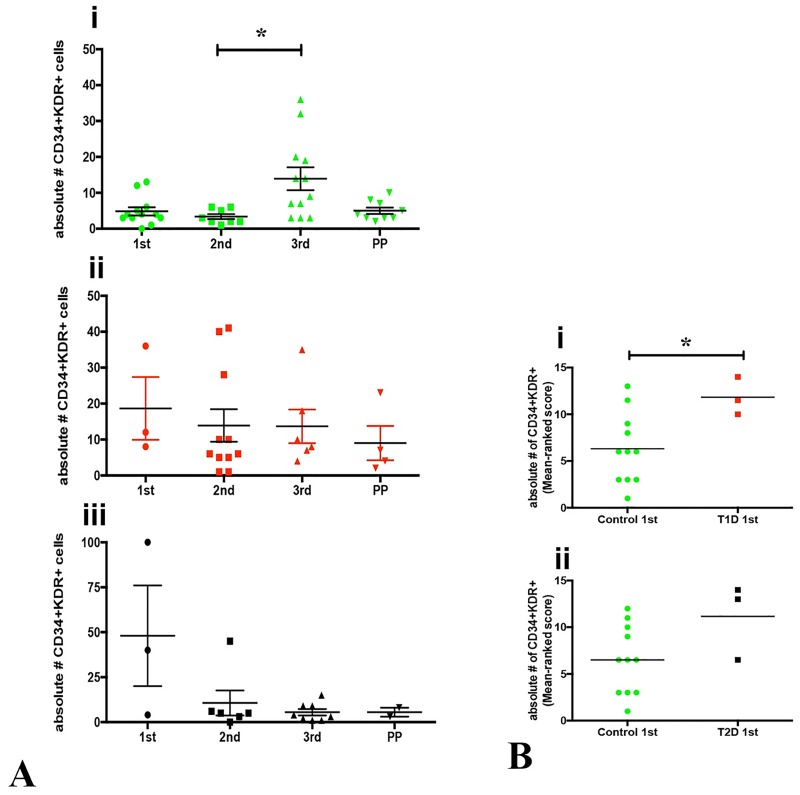
Total CD34+KDR+ cells across pregnancy and postpartum in control, type 1 and type 2 diabetic women. **(A**) Absolute numbers (per 10^5^ mononuclear cells) of circulating CD45dimCD34+SSlowKDR+ (CD34+KDR+) cells across pregnancy and postpartum from control (**i**), type 1 (**ii**) and type 2 (**iii**) diabetic women. In control women, CD34+KDR+ cells were elevated in 3^rd^ trimester and significantly higher compared to 2^nd^ trimester (p = 0.013; **i**). Such fluctuation was not observed in type 1 and type 2 diabetic women (**i, ii**). (**B)** Comparisons between control (green) and type 1 (red) (**i**) and type 2 diabetes (black) (**ii)** at 1^st^ trimester demonstrated that type 1 (p = 0.033), but not type 2 diabetic women (p = 0.090) had significantly more CD34+KDR+ CACs than control women. No differences were found when comparing control and diabetic (type 1 and type 2) women at 2^nd^, 3^rd^ trimester and postpartum stages. Data in **A** were compared using Kruskal-Wallis test; **B** using Mann-Whitney test, considering p<0.05. Data in **Bi** and **Bii** were shown as a rank score graphic.

The frequency of total CD34+KDR+ CACs (sum of CD133+KDR+ and CD133-KDR+) was elevated at 3^rd^ trimester in control women, and significantly higher than in 2^nd^ trimester (Fig 4Ai; p = 0.013). These data corroborate the results of others who measured CD34+KDR+ cell number or frequency during pregnancy [11, 13]. Interesting, in type 1 and type 2 diabetes, no change in frequency or number of these cells was observed over pregnancy (Fig 4Aii and 4Aiii). First trimester circulatory responses may be critical in diabetic women, since CD34+KDR+ CACs were elevated versus controls. Type 1 diabetic women had significantly more CD34+KDR+ cells at 1^st^ trimester compared to control (Fig 4Bi; p = 0.033); CD34+KDR+ cells also increased in type 2 diabetic women, but not a statistically different level (Fig 4Bii; p = 0.090).

**Fig 5:**
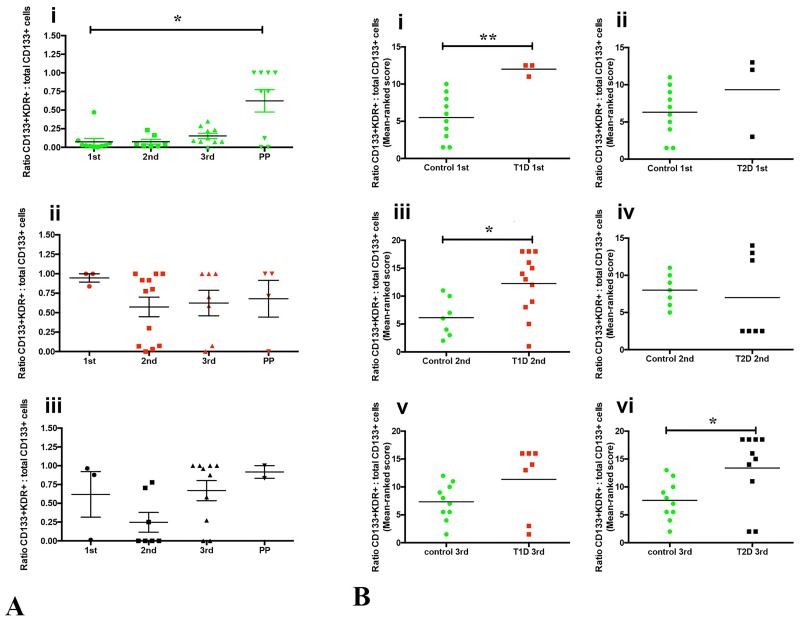
CD133+KDR+ to total CD133+ cell ratios across pregnancy and postpartum in control, type 1 and type 2 diabetic women. **(A**) Ratio of CD45dimCD34+SSlowCD133+KDR+ (CD133+KDR+) to total CD45dimCD34+SSlowCD133+ (CD133+) cells across pregnancy and postpartum from control (**i**), type 1 (**ii**) and type 2 (**iii**) diabetic women. In control women, the CD133+KDR+/ CD133+ ratio revealed that the proportion of CD133+KDR+ is relatively low during pregnancy (1^st^, 2^nd^ and 3^rd^ trimester) compared to non-pregnant women (postpartum). CD133+KDR+/ CD133+ ratio at 1^st^ trimester in control women was statistically lower compared to postpartum (p = 0.047; **i**). This ratio was relatively higher in type 1 **(ii)** and type 2 **(iii)** diabetes compared to control. In **(B),** comparisons between diabetic and control women identified that CD133+KDR+/CD133+ cell ratios were higher in pregnant but not postpartum diabetics. In type 1 diabetic women, significant differences were present in 1^st^ (p = 0.007; **Bi**) and 2^nd^ (p = 0.021; **Biii**) trimester, but not in 3^rd^ trimester (p = 0.113; **Bv**). In type 2 diabetic women, 1^st^ (p = 0.272; **Bii**) and 2^nd^ (p = 0.688; **Biv**) trimesters were not significantly different but 3^rd^ trimester was (p = 0.026; **Bvi**). Data in **A** were compared using Kruskal-Wallis test; **B** using Mann-Whitney test, considering p<0.05. Data in **Bi** and **Bii** were shown as a rank score graphic.

The ratio of CD133+KDR+ (CD34+CD45dimSSlowCD133+KDR+) to total CD133+ (CD34+CD45dimSSlowCD133+) was analysed to compare KDR acquisition by CD133+ CACs over normal and diabetic pregnancies. Postpartum control women, but not type 1 or type 2 diabetic women, had a higher ratio of CD133+KDR+/total CD133+ cells compared to pregnant women (1^st^, 2^nd^ and 3^rd^ trimesters). In control women, this ratio was significantly different at 1^st^ trimester compared to non-pregnant (postpartum) (Fig 5Ai–5Aiii; **p = 0.047**). Comparing type 1 and type 2 diabetic women with control, the CD133+KDR+/CD133+ cell ratios were higher in type 1 diabetic women at 1^st^ and 2^nd^ trimester compared to control women (p = 0.007 and p = 0.020, respectively; Fig 5Bi–5Biv). At 3^rd^ trimester, the CD133+KDR+/CD133+ cell ratio was higher in both type 1 and type 2 diabetic women (Fig 5Bv and 5Bvi), but statistically significant only for type 2 diabetic women compared to controls (Fig 5Bvi; p = 0.026). Type 1 diabetes did not influence the postpartum CD133+KDR+/CD133+ cell ratio compared to controls.

**Fig 6:**
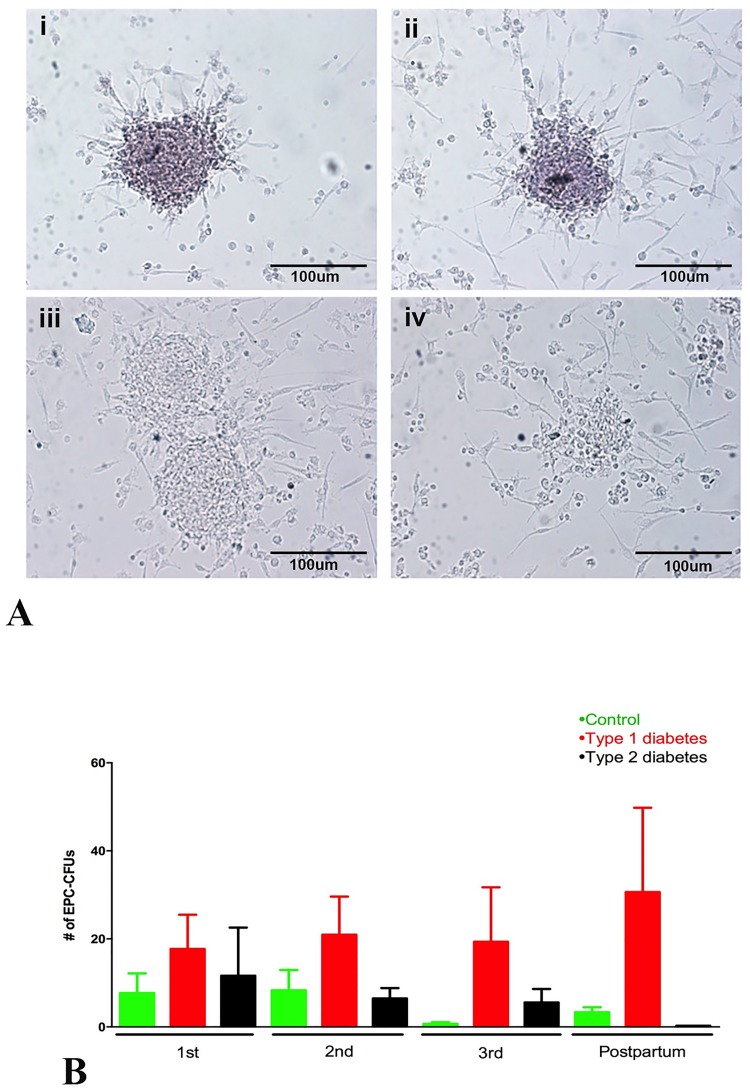
Early outgrowth colonies. **(A)** Examples of early outgrowth colonies (**i, ii** and **iii)** and a cell cluster that was not scored as a colony (**iv**). The colonies had a central core of round cells, with more elongated sprouting cells at the periphery. **(B)** Quantification of early outgrowth colonies demonstrated that diabetes (type 1: red bars; type 2: black bars) did not significantly affect the ability of CACs to form colonies compared to control women (green bars). Data were compared using Kruskal-Wallis test, considering p<0.05. Bars: 100um.

Early outgrowth colonies (CFU-Hill) did not differ significantly in number across pregnancy and postpartum within any gestational group or between the groups (Fig 6).

## Discussion

The major findings in this study were that preconception diabetes is associated with failure to respond to pregnancy-driven elevations in CPCs (CD34+CD45dimSSlow cells) and CACs (CD133+KDR+ and CD133+KDR- cells). Our findings are summarized in Table 4. Differences in frequencies of CD133+KDR- CACs between diabetic and normal women appeared to be gestationally-restricted because they did not persist postpartum (non-pregnant state). These data suggest that even in diabetic women with good glycemic control, diabetes alters CPC and CACs levels. This may be due to failure of CPC/CAC mobilization into circulation, defective maturation (i.e. acquisition of KDR expression) or more rapid consumption of CACs during maternal vascular adaptions to pregnancy. Such changes would be expected to increase risks for blood vessel-related pregnancy complications, such as preeclampsia.

**Table 4:** Summary of differences between diabetes and matched controls in CPC and CAC levels.

	Type 1 diabetes	Type 2 diabetes
**1**^**st**^ **trimester**	**increased** CD34+KDR+	**increased** CD34+CD133+KDR+
	**increased** CD34+CD133+KDR+/ total CD34+CD133+	
**2**^**nd**^ **trimester**	**increased** CD34+CD133+KDR+/ total CD34+CD133+	**decreased** CD34+CD133+KDR-
**3**^**rd**^ **trimester**	**decreased** CD34+CD45dimSSlow	**decreased** CD34+CD133+KDR-
		**increased** CD34+CD133+KDR+/ total CD34+CD133+

The sequence of physiological changes that are induced within the maternal cardiovascular system during pregnancy suggest that several different waves of CACs recruitment from marrow to blood may be needed for gestational remodeling of different target organs. The first potential driver for CACs recruitment would be the significant expansion in plasma volume that occurs very early in human pregnancy under hormonal control. Chapman and colleagues [30] studied normal women intending to conceive during their pre-conception, menstrual cycles and at 6, 8, 10, 12 and 24 weeks post-conception. Decreased vascular resistance increased cardiac output and declining mean arterial pressure began after ovulation and prior to placental formation [30]. Between the ovulatory phase of the cycle and gestation week 6, plasma estradiol, a molecule known to mobilize CACs (CD34+KDR+ cells) [14], rose tenfold. A rise in effective renal plasma flow consistent with primary renal vasodilation was also significant by gestation week 6 [30]. During these early pregnancy weeks, the uterine lining also transforms from cycling endometrium into decidua, a process that involves extensive neoangiogenesis. The next critical angiogenic events are those that support placental development between 6 and 12 weeks of gestation. Morphological and ultrasonographic studies of human implantation sites at 8–16 weeks after conception [31, 32] report rapid hemodynamic changes within decidua and maternal spiral arteries that feed into the placental intervillous space where maternal-fetal nutrient/waste exchange occurs. Following completion of the embryonic phase (week 8 of gestation), fetal growth becomes a new driver of maternal gestational angiogenesis and by the end of the 2^nd^ trimester, typical human fetuses weigh 820 g—1000 g which is 3x more than their placenta (280 g–300g). Although the placental weight doubles over 3^rd^ trimester, a typical late pregnancy fetus (2500 g– 3000 g) is 6-7x heavier than its placenta (400 g–500 g) [33]. Maternal uterine vascular expansion must be needed for healthy support of this terminal phase of rapid fetal growth. In addition, stromal cell-derived factor 1 alpha (SDF1*α*), a crucial factor in recruitment of bone-marrow derived CD34+ cells [34] is elevated in maternal plasma during pregnancy. Levels are slightly reduced between 1^st^ and 2^nd^ trimesters then dramatically escalate between 2^nd^ and 3^rd^ trimester suggesting variable needs for CACs mobilization over gestation [35]. Changes in maternal plasma concentrations are largely due to secretions from conceptus-derived placental trophoblasts [36]. This is expected to create a concentration gradient that would preferentially localize CACs to the maternal-fetal interface.

In type 1 and type 2 diabetic women, the frequency of circulating CD34+CD45dimSSlow cells was invariant in across pregnancy and similar to postpartum. This was in stark contrast to normal women whose CD34+CD45dimSSlow cells were elevated across all trimesters of gestation, particularly in trimesters one and three. Increase in CPCs and CACs have been reported by others during normal pregnancy [12, 13, 16], although gestational age ranges, antibody reagents and cell phenotyping vary between the reports which has made understating of CPCs/CACs in pregnancy difficult. Diabetes additionally alters the balance between CD133+KDR- and CD133+KDR+ CAC subsets across gestation. In 1st trimester, type 2 diabetic women had very high circulating numbers of CD133+KDR+ CACs, suggesting dysregulated maturation of CACs or limited recruitment of CD133+KDR+ CACs to organs undergoing the rapid vascular changes of early pregnancy. Since acquisition of KDR expression is part of CACs maturation/differentiation, the lower frequencies of CD133+KDR- cells in these patients at 2nd and 3rd trimester also supports the postulate of accelerated CAC differentiation in pregnant type 2 diabetic women.

When total CD34+KDR+ CACs (the sum of CD133+KDR+ and CD133-KDR+) were analyzed, we found that CD34+KDR+ CACs were significantly increased in normal pregnancy in 3^rd^ trimester as previously reported by Luppi and colleagues [12]. KDR expressing CACs were at much lower levels than CD34+CD133+KDR- CACs in non-pregnant women and women in 1^st^ or 2^nd^ trimester. In type 1 diabetic women, however, an abnormal elevation of CD34+KDR+ CACs was detected in 1^st^ trimester compared to control. CD34+KDR+ CACs are markers of vascular injury although the mechanisms for their increases or decreases in the circulation of patients with cardiovascular diseases are unclear. In coronary artery disease, fewer CD34+KDR+ cells are seen [37, 38] while in heart failure or atherosclerotic disease progression more CD34+KDR+ cells occur in circulation [39, 40]. Similarly, in type 2 diabetic patients Fadini and colleagues [41] reported that KDR+ CACs are severely reduced in those with peripheral arterial disease but elevated in those with diabetic retinopathy. This “diabetic paradox” phenomenon may apply to our finding that preconception diabetes differentially altered CD133+ and KDR+ CAC numbers in a gestation stage, specific manner. The dynamic physiological changes of pregnancy (hormones, cytokines, angiokines, growth factors, etc.) may differentially modulate the frequency of CACs by controlling balances between CAC differentiation, recruitment and target tissue consumption.

In contrast to CD133+KDR+ CACs, CD133+KDR- CACs in 1^st^ trimester of type 1 and type 2 diabetic women were at statistically comparable levels to control women. In 2^nd^ and 3^rd^ trimester of type 2 diabetic pregnancies however, CD133+KDR- CACs were remarkably deficient. Mechanisms to account for this were not addressed but may include failure of differentiation in marrow, failure of mobilization into circulation or excessive consumption in target organs, particularly the gravid uterus. While lower absolute numbers of CD133+KDR- CACs were also present in 2^nd^ and 3^rd^ trimesters of type 1 diabetic women, these differences did not reach statistical significance in comparison to either control or type 2 diabetic women.

KDR+ CACs are more differentiated than KDR- CACs and this subset responds to VEGF. To better understand the dynamic relationships between these subsets, CD133+KDR+/ total CD133+ ratios were calculated that strongly suggested the balance between the two subsets was altered by preconception diabetes. While women with type 2 diabetes had fewer CD133+KDR- CACs at 2^nd^ and 3^rd^ trimester and higher CD133+KDR+ CACs at 1^st^ trimester compared to control women, the CD133+KDR+/ CD133+ ratio demonstrated that diabetes elevates the frequency of CD133+KDR+ CACs in all stages of pregnancy in type 1 and type 2 diabetes. This finding is particularly important since control pregnant women had low levels of CD133+KDR+ cells compared to CD133+KDR- CACs, demonstrating that a coordinate recruitment and of CACs are required in pregnancy.

Pregnancy is a physiological state during which the systemic endothelium is continuously exposed to changing concentrations and compositions of circulating factors that maintain gestational success. Many of these factors also participate in CACs differentiation, recruitment into circulation and exodus from circulation. Examples of these regulatory molecules include VEGF, placental growth factor (PGF), granulocyte colony-stimulating factor (G-CSF) and granulocyte- macrophage colony-stimulating factor (GM-CSF) [42–44]. Progenitor cell mobilopathy has been characterized in diabetes and therapies based on G-CSF and GM-CSF are already in use to optimize stem-cell peripheralization and CAC recruitment into circulation [1, 45]. CAC mobilization from marrow is a complex process, regulated by a network of factors well beyond individual cytokine deficits. Further studies will be necessary to understand the physiological, pregnancy-associated drivers of CACs recruitment from marrow to blood and how these are deviated in women who are diabetic prior to conception. The apparent return of CAC numbers to control levels after pregnancy suggests that pregnancy itself does not enhance long-term negative circulatory outcomes for diabetic mothers. A much larger study would be needed to assess correlations between specific gestational pathologies and the disturbances in CAC frequencies documented in this pilot study.

## References

[1] Fadini GP, Ferraro F, Quaini F, Asahara T, Madeddu P. Concise review: diabetes, the bone marrow niche, and impaired vascular regeneration. Stem Cells Transl Med. 2014; 3(8): 949–957. 10.5966/sctm.2014-0052 24944206 PMC4116251

[2] Asahara T, Murohara T, Sullivan A, Silver M, van der Zee R, Li T, et al Isolation of putative progenitor endothelial cells for angiogenesis. Science. 1997; 275(5302): 964–967. 9020076 10.1126/science.275.5302.964

[3] Jaipersad AS, Lip GY, Silverman S, Shantsila E. The role of monocytes in angiogenesis and atherosclerosis. J Am Coll Cardiol. 2014; 7–14; 63(1):1–11. 10.1016/j.jacc.2013.09.019 24140662

[4] Hill JM, Zalos G, Halcox JP, Schenke WH, Waclawiw MA, Quyyumi AA, et al Circulating endothelial progenitor cells, vascular function, and cardiovascular risk. N Engl J Med. 2003; 13; 348 (7): 593–600. 10.1056/NEJMoa022287 12584367

[5] Osol G, Mandala M. Maternal Uterine Vascular Remodeling During Pregnancy. Physiology (Bethesda) 2009; 24: 58–71. 19196652 10.1152/physiol.00033.2008 PMC2760472

[6] Lima PDA, Zhang J, Dunk C, Lye SL, Croy BA. Leukocyte driven-decidual angiogenesis in early pregnancy. Cell Mol Immunol. 2014; 11(6): 522–537. 10.1038/cmi.2014.63 25066422 PMC4220841

[7] Sanghavi M, Rutherford JD. Cardiovascular physiology of pregnancy. Circulation. 2014; 130(12): 1003–1008. 10.1161/CIRCULATIONAHA.114.009029 25223771

[8] Flo K, Blix ES, Husebekk A, Thommessen A, Uhre AT, Wilsgaard T, et al A longitudinal study of maternal endothelial function, inflammatory response and uterine artery blood flow during the second half of pregnancy. Acta Obstet Gynecol Scand. 2015; 95(2): 225–232. 10.1111/aogs.12802 26462064

[9] Lash GE, Schiessl B, Kirkley M, Innes BA, Cooper A, Searle RF, et al Expression of angiogenic growth factors by uterine natural killer cells during early pregnancy. J Leukoc Biol. 2006; 80(3): 572–80. 10.1189/jlb.0406250 16816146

[10] Lash GE, Pitman H, Morgan HL, Innes BA, Agwu CN, Bulmer JN. Decidual macrophages: key regulators of vascular remodeling in human pregnancy. J Leukoc Biol. 2016; 100(2): 315–25. 10.1189/jlb.1A0815-351R 26819320

[11] Hubel CA, Sipos PI, Crocker IP. Endothelial progenitor cells. Their potential role in pregnancy and preeclampsia. Pregnancy Hypertens. 2011; 1(1): 48–58. 10.1016/j.preghy.2010.11.001 26104231

[12] Luppi P, Powers RW, Verma V, Edmunds L, Plymire D, Hubel CA. Maternal circulating CD34+VEGFR-2+ and CD133+VEGFR-2+ progenitor cells increase during normal pregnancy but are reduced in women with preeclampsia. Reprod Sciences. 2010; 17: 643–652. 10.1177/1933719110366164 PMC2893245 20360595

[13] Buemi M, Allegra A, D'Anna R, Coppolino G, Crascì E, Giordano D, et al Concentration of circulating endothelial progenitor cells (EPC) in normal pregnancy and in pregnant women with diabetes and hypertension. Am J Obstet Gynecol. 2007; 196(1): 68e1–6. 10.1016/j.ajog.2006.08.032 17240239

[14] Bulut D, Albrecht N, Imöhl M, Günesdogan B, Bulut-Streich N, Börgel J, et al Hormonal status modulates circulating endothelial progenitor cells. Clin Res Cardiol. 2007; 96(5): 258–63. 10.1007/s00392-007-0494-z 17323014

[15] Parsanezhad ME, Attar A, Namavar-Jahromi B, Khoshkhou S, Khosravi-Maharlooei M, Monabati A, et al Changes in endothelial progenitor cell subsets in normal pregnancy compared with preeclampsia. J Chin Med Assoc. 2015; 78(6): 345–352. 10.1016/j.jcma.2015.03.013 26006732

[16] Acosta JC, Haas DM, Saha CK, Dimeglio LA, Ingram DA, Haneline LS. Gestational diabetes mellitus alters maternal and neonatal circulating endothelial progenitor cell subsets. Am J Obstet Gynecol. 2011; 204(3): 254.e8–254.e15. 10.1016/j.ajog.2010.10.913 PMC3057499 21167470

[17] Karumanchi SA, Granger JP. Preeclampsia and Pregnancy-Related Hypertensive Disorders. Hypertension. 2016; 67(2): 238–42. 10.1161/HYPERTENSIONAHA.115.05024 26693822 PMC4755281

[18] Evers IM, de Valk HW, Visser GH. Risk of complications of pregnancy in women with type 1 diabetes: Nationwide prospective study in the Netherlands. BMJ 2004; 328: 915 10.1136/bmj.38043.583160.EE 15066886 PMC390158

[19] Taylor R, Davison JM. Type 1 diabetes and pregnancy. BMJ 2007; 334: 742–745. 10.1136/bmj.39154.700417.BE 17413175 PMC1847857

[20] Holmes VA, Young IS, Patterson CC, Maresh MJA, Pearson DWM, Walker JD, et al Diabetes and Preeclampsia Intervention Trial (DAPIT) Study Group. Preeclampsia Intervention Trial (DAPIT) Study Group The Role of Angiogenic and Antiangiogenic Factors in the Second Trimester in the Prediction of Preeclampsia in Pregnant Women With Type 1 Diabetes. Diabetes Care. 2013; 36(11): 3671–3677. 23920083 10.2337/dc13-0944 PMC3816852

[21] Howangyin KY, Silvestre JS. Diabetes mellitus and ischemic diseases: molecular mechanisms of vascular repair dysfunction. Arterioscler Thromb Vasc Biol. 2014; 34(6): 1126–1135. 10.1161/ATVBAHA.114.303090 24675660

[22] Maiorino MI, Casciano O, Volpe ED, Bellastella G, Giugliano D, Esposito K. Reducing glucose variability with continuous subcutaneous insulin infusion increases endothelial progenitor cells in type 1 diabetes: an observational study. Endocrine. 2015; 52(2): 244–252. 10.1007/s12020-015-0686-7 26184417

[23] Sorrentino AS, Bahlmann FH, Besler C, Müller M, Schulz S, Kirchhoff N, et al Oxidant stress impairs in vivo reendothelialization capacity of endothelial progenitor cells from patients with type 2 diabetes mellitus: restoration by the peroxisome proliferator-activated receptor gamma agonist rosiglitazone. Circulation. 2007; 116: 163–173. 10.1161/CIRCULATIONAHA.106.684381 17592079

[24] Churdchomjan W, Kheolamai P, Manochantr S, Tapanadechopone P, Tantrawatpan C, U-Pratya Y, et al Comparison of endothelial progenitor cell function in type 2 diabetes with good and poor glycemic control. BMC Endocr Disord. 2010; 10: 5 10.1186/1472-6823-10-5 20374643 PMC2858721

[25] Hernandez SL, Gong JH, Chen L, Wu IH, Sun JK, Keenan HA, et al Characterization of circulating and endothelial progenitor cells in patients with extreme-duration type 1 diabetes. Diabetes Care. 2014; 37: 2193–2201. 10.2337/dc13-2547 24780357 PMC4113171

[26] Fadini GP, Miorin M, Facco M, Bonamico S, Baesso I, Grego F, et al Circulating endothelial progenitor cells are reduced in peripheral vascular complications of type 2 diabetes mellitus. J Am Coll Cardiol. 2005; 3: 45(9): 1449–1457. 10.1016/j.jacc.2004.11.067 15862417

[27] Rigato M, Bittante C, Albiero M, Avogaro A, Fadini GP. Circulating Progenitor Cell Count Predicts Microvascular Outcomes in Type 2 Diabetic Patients. J Clin Endocrinol Metab. 2015; 100(7): 2666–2672. 10.1210/jc.2015-1687 25942480

[28] Fadini GP, Sartore S, Albiero M, Baesso I, Murphy E, Menegolo M, et al Number and function of endothelial progenitor cells as a marker of severity for diabetic vasculopathy. Arterioscler Thromb Vasc Biol. 2006; 26(9): 2140–2146. 10.1161/01.ATV.0000237750.44469.88 16857948

[29] Schmidt-Lucke C, Rossig L, Fichtlscherer S, Vasa M, Britten M, Kämper U, et al Reduced Number of Circulating Endothelial Progenitor Cells Predicts Future Cardiovascular Events: Proof of Concept for the Clinical Importance of Endogenous Vascular Repair. Circulation 2005; 111: 2981–2987. 10.1161/CIRCULATIONAHA.104.504340 15927972

[30] Chapman AB, Abraham WT, Zamudio S, Coffin C, Merouani A, Young D, et al Temporal relationships between hormonal and hemodynamic changes in early human pregnancy. Kidney Int. 1998; 54(6): 2056–63. 10.1046/j.1523-1755.1998.00217.x 9853271

[31] Pijnenborg R, Bland JM, Robertson WB, Brosens I. Uteroplacental arterial changes related to interstitial trophoblast migration in early human pregnancy. Placenta. 1983; 4:397–414. 6634666 10.1016/s0143-4004(83)80043-5

[32] Pijnenborg R, Vercruysse L, Hanssens M. The uterine spiral arteries in human pregnancy: facts and controversies. Placenta. 2006; 27 (9–10): 939–58. 10.1016/j.placenta.2005.12.006 16490251

[33] Faye Petersen O, Heller D, Joshi V. Handbook of placental pathology. 2006; London, New York: Taylor and Francis.

[34] Jo DY, Rafii S, Hamada T, Moore MA. Chemotaxis of primitive hematopoietic cells in response to stromal cell-derived factor-1. J Clin Invest. 2000; 105(1): 101–11. 10.1172/JCI7954 10619866 PMC382585

[35] Martínez-Varea A, Pellicer B, Serra V, Hervás-Marín D, Martínez-Romero A, Bellver J, et al The Maternal Cytokine and Chemokine Profile of Naturally Conceived Gestations Is Mainly Preserved during In Vitro Fertilization and Egg Donation Pregnancies. J Immunol Res. 2015; 2015:128616 10.1155/2015/128616 26346343 PMC4546760

[36] Wu X, Jin LP, Yuan MM, Zhu Y, Wang MY, Li DJ. Human first-trimester trophoblast cells recruit CD56brightCD16- NK cells into decidua by way of expressing and secreting of CXCL12/stromal cell-derived factor 1. J Immunol. 2005; 175(1): 61–8. 15972632 10.4049/jimmunol.175.1.61

[37] Briguori C, Testa U, Riccioni R, Colombo A, Petrucci E, Condorelli G, et al Correlations between progression of coronary artery disease and circulating endothelial progenitor cells. FASEB J. 2010; 24: 1981–1988. 10.1096/fj.09-138198 20056714

[38] Liguori A, Fiorito C, Balestrieri ML, Crimi E, Bruzzese G, Williams-Ignarro S, et al Functional impairment of hematopoietic progenitor cells in patients with coronary heart disease. Eur J Haematol. 2008; 80 258–264. 10.1111/j.1600-0609.2007.01007.x 18081701

[39] Valgimigli M, Rigolin GM, Fucili A, Porta MD, Soukhomovskaia O, Malagutti P, et al CD34+ and endothelial progenitor cells in patients with various degrees of congestive heart failure. Circulation. 2004;110(10):1209–1212. 10.1161/01.CIR.0000136813.89036.21 15249502

[40] Schmidt-Lucke C, Rossig L, Fichtlscherer S, Vasa M, Britten M, Kamper U, et al Reduced number of circulating endothelial progenitor cells predicts future cardiovascular events. Circulation 2005; 111: 2981–2987. 10.1161/CIRCULATIONAHA.104.504340 15927972

[41] Fadini GP, Sartore S, Baesso I, Lenzi M, Agostini C, Tiengo A, et al Endothelial progenitor cells and the diabetic paradox. Diabetes Care. 2006; 29(3): 714–6. 16505536 10.2337/diacare.29.03.06.dc05-1834

[42] Perricone R, De Carolis C, Giacomelli R, Guarino MD, De Sanctis G, Fontana L. GM-CSF and pregnancy: evidence of significantly reduced blood concentrations in unexplained recurrent abortion efficiently reverted by intravenous immunoglobulin treatment. Am J Reprod Immunol. 2003; 50(3):232–7. 14629028 10.1034/j.1600-0897.2003.00083.x

[43] Li B, Sharpe EE, Maupin AB, Teleron AA, Pyle AL, Carmeliet P, et al VEGF and PlGF promote adult vasculogenesis by enhancing EPC recruitment and vessel formation at the site of tumor neovascularization. FASEB. 2006; 20(9): 1495–7. 10.1096/fj.05-5137fje 16754748

[44] Scarpellini F and Sbracia M. Use of granulocyte colony-stimulating factor for the treatment of unexplained recurrent miscarriage: a randomised controlled trial. Human Reproduction. 2009; 24 (11): 2703–2708. 10.1093/humrep/dep240 19617208

[45] DiPersio JF. Diabetic stem-cell "mobilopathy". N Engl J Med. 2011; 365(26): 2536–2538. 10.1056/NEJMcibr1112347 22204729

